# **Preoperative MRI-based prediction of autograft diameter in ACL reconstruction using hamstring and peroneus longus tendons**

**DOI:** 10.1186/s13018-026-06919-9

**Published:** 2026-05-03

**Authors:** Aashay Prasad Pande, Atmananda S. Hegde, K. Suprasanna, Chethan B. Shetty, Prajwal Prabhudev Mane, Mayur Narayanchandra Hebsur

**Affiliations:** 1 Department of Orthopaedics, Kasturba Medical College Mangalore, Manipal Academy of Higher Education, Manipal, Karnataka 576 104 India; 2 Department of Radiodiagnosis and Imaging, Kasturba Medical College Mangalore, Manipal Academy of Higher Education, Manipal, Karnataka 576104 India; 3 Department of Orthopaedics, Royal Infirmary Hospital and Woodend Hospital, Aberdeen, Scotland, UK; 4 Department of Orthopaedics, Sanjay Gandhi Institute of Trauma and Orthopaedics, Bangalore, India; 5 Department of Orthopaedics, Kasturba Medical College Mangalore, Manipal Academy of Higher Education, Manipal, Karnataka 575 104 India

**Keywords:** Anterior cruciate ligament, Tendon autograft, Cross-sectional area, Magnetic resonance imaging

## Abstract

**Background:**

To evaluate the predictive value of magnetic resonance imaging (MRI)-derived cross-sectional area (CSA) measurements of hamstring and peroneus longus tendons in estimating intraoperative autograft diameter during anterior cruciate ligament (ACL) reconstruction, and to identify clinically useful CSA cut-offs for achieving graft diameters ≥ 8 mm.

**Methods:**

Fifty-two patients undergoing primary ACL reconstruction were included in this prospective observational study, with 26 each in the hamstring tendon (HT) and peroneus longus tendon (PLT) autograft groups. Preoperative CSA was measured on axial MRI at specific anatomical levels. Intraoperative graft diameters were recorded post- harvest. Correlation analysis and receiver operating characteristic curves were used to assess predictive value and determine CSA thresholds for adequate graft size.

**Results:**

For the HT group, CSA measured at the medial femoral condyle showed the strongest correlation with intraoperative diameter (*r* = 0.782; *p* < 0.001), with an optimal CSA cut-off of ≥ 19.15 mm² (AUC 0.917, sensitivity: 85%, specificity: 100%). For the PLT group, ankle-level CSA best predicted graft diameter (cut-off: ≥12.84 mm², AUC 0.958, sensitivity: 95.8%, specificity: 100%).

**Conclusion:**

MRI-based CSA measurement provides a reliable, non-invasive method for predicting autograft adequacy. This study establishes clinically relevant CSA thresholds for HT and PLT tendons and is the first to report such values for PLT, aiding preoperative graft selection in ACL reconstruction.

## Background

Anterior cruciate ligament (ACL) injuries are among the most common knee traumas, particularly affecting individuals engaged in athletic and high-demand physical activities. The global incidence ranges from 30 to 78 cases per 100,000 persons annually, with a higher prevalence in younger, active populations [1]. ACL reconstruction (ACLR) remains the gold standard treatment to restore joint stability and reduce the risk of long-term sequelae such as osteoarthritis [2–3].

The success of ACLR is significantly influenced by graft selection and size. Autografts including hamstring tendons (HT), peroneus longus tendon (PLT), quadriceps tendon (QT), and bone–patellar tendon–bone (BPTB) are commonly used [4–9]. Among these, ensuring a graft diameter of at least 8 mm has been associated with improved biomechanical strength and lower failure rates [10–16]. Consequently, preoperative prediction of graft adequacy is vital for surgical planning, especially to avoid intraoperative surprises and reduce reliance on backup grafts [17–19].

Magnetic resonance imaging (MRI) has emerged as a valuable, non-invasive modality for estimating graft dimensions preoperatively [13, 17, 20–21]. While various MRI-based prediction models exist for hamstring tendons [13, 17], data on the peroneus longus tendon, a graft gaining popularity due to favourable biomechanical properties and lower donor-site morbidity [22–23] remain limited. Notably, recent studies have demonstrated comparable outcomes between PLT and HT autografts, with PLT offering potential advantages in graft size and postoperative muscle preservation [22, 24–26].

Despite increasing clinical interest, a validated imaging-based method to predict PLT graft diameter remains underexplored. Moreover, reliable anatomical cut-off values based on cross-sectional area (CSA) measurements that correlate with intraoperative graft size are yet to be standardized.

Therefore, the present study aimed to assess the predictive value of MRI-derived CSA measurements of the peroneus longus and hamstring tendons in estimating intraoperative autograft diameter. The study further sought to establish clinically useful CSA cut-offs for predicting grafts with adequate thickness (≥ 8 mm) in patients undergoing primary ACL reconstruction.

## Methods

### Study design and participants

This prospective observational study was conducted at a tertiary care teaching hospital for a duration of 2 years from June 2023 to June 2025 following approval by the Institutional Scientific and Ethics Committee. Fifty-two patients undergoing primary anterior cruciate ligament (ACL) reconstruction were enrolled and allocated into two autograft groups: hamstring tendon (HT; *n* = 26) and peroneus longus tendon (PLT; *n* = 26).

For the hamstring autograft group, a sample size of *n* = 26 was calculated based on a reported correlation coefficient of *r* = 0.64 from the study of Hollnagel et al. [13]. For the peroneus longus group, no prior correlation data was available in the literature; therefore, a moderate correlation of *r* = 0.5 was assumed to estimate a comparable sample size of *n* = 26.

Inclusion criteria were skeletally mature individuals with isolated ACL tears confirmed clinically and radiologically, scheduled for primary ACL reconstruction.

Exclusion criteria included:

- Revision ACL surgery.
- Prior surgery on the ipsilateral knee.
- Multi-ligamentous knee injuries.
- Concomitant bony injuries around the knee.
- Congenital or acquired musculoskeletal deformities.
- Suboptimal or incomplete MRI imaging.
- MRI contraindications.
- Patients who declined consent.

All participants provided written informed consent.

### MRI acquisition and data collection

All imaging was performed using a 1.5-Tesla MRI scanner with standardized knee and ankle protocols. A single experienced musculoskeletal radiologist conducted and interpreted all MRI measurements using digital callipers within the RadiAnt DICOM viewer software to ensure consistency and eliminate inter-observer variability.

### MRI assessment of hamstring tendons

**Fig. 1:**
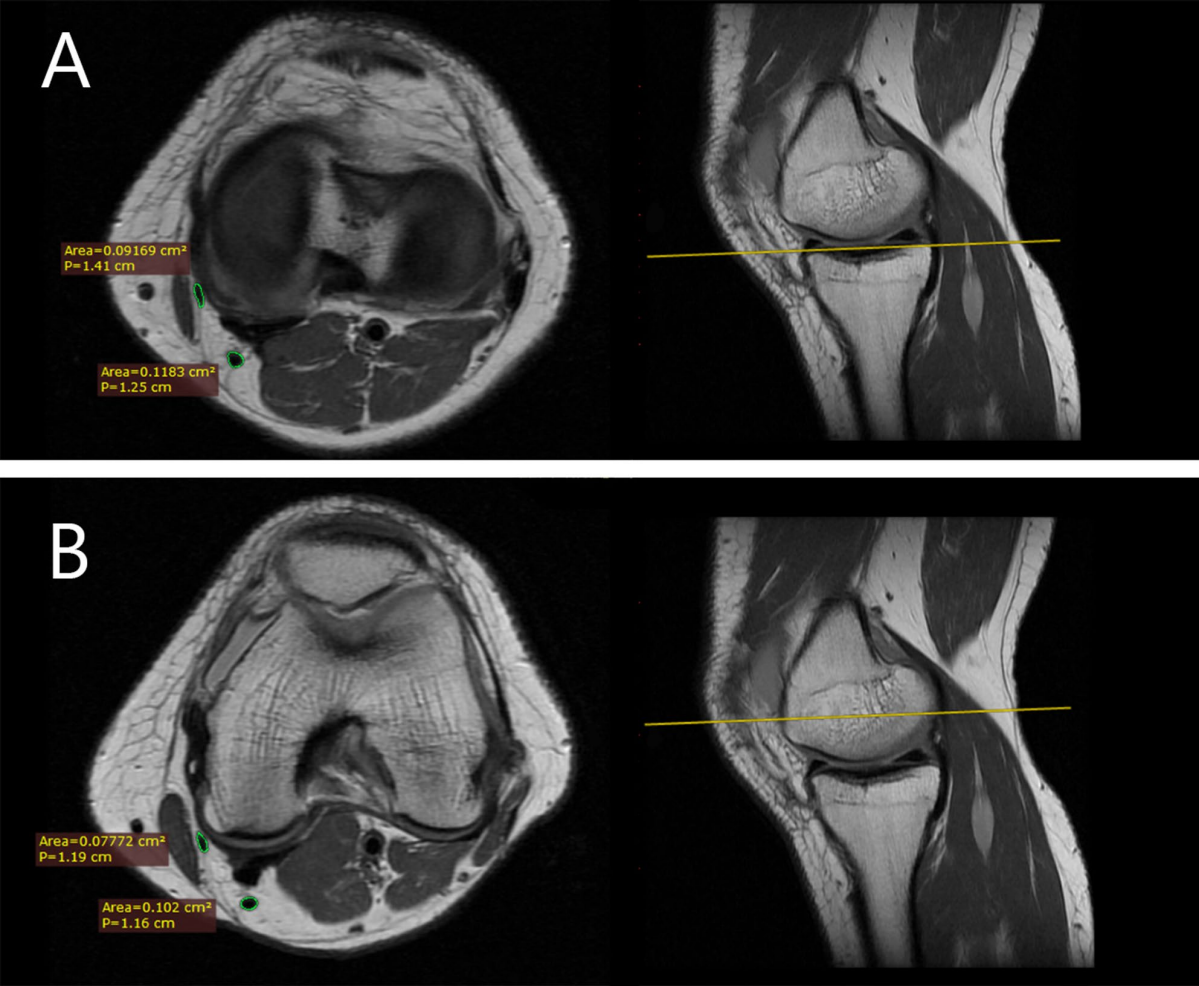
MRI images (axial image with sagittal reference for localisation) showing CSA measurement of the hamstring tendons (semitendinosus + gracilis) at the levels of the knee joint (**A**) and the maximum AP diameter of medial femoral condyle (**B**)

For patients in the HT group, the same knee MRI used to diagnose the ACL tear was also utilized for tendon CSA analysis. The semitendinosus (ST) and gracilis (GT) tendons were assessed on preoperative sagittal and axial sequences. CSA measurements were obtained at two anatomical levels (Fig. 1):

1. At the level of the knee joint line.
2. At the level of maximum anteroposterior (AP) diameter of the medial femoral condyle.

Axial slices were referenced against sagittal views to ensure anatomical accuracy. These two anatomical levels—the knee joint line and the point of maximum anteroposterior diameter of the medial femoral condyle—were selected because they offer clear, reproducible anatomical landmarks on MRI [27–28]. Previous studies have demonstrated that CSA measured at these levels shows strong correlation with intraoperative graft diameter, making them reliable and clinically relevant points for prediction [13–14, 17].

### MRI assessment of the peroneus longus tendon

**Fig. 2:**
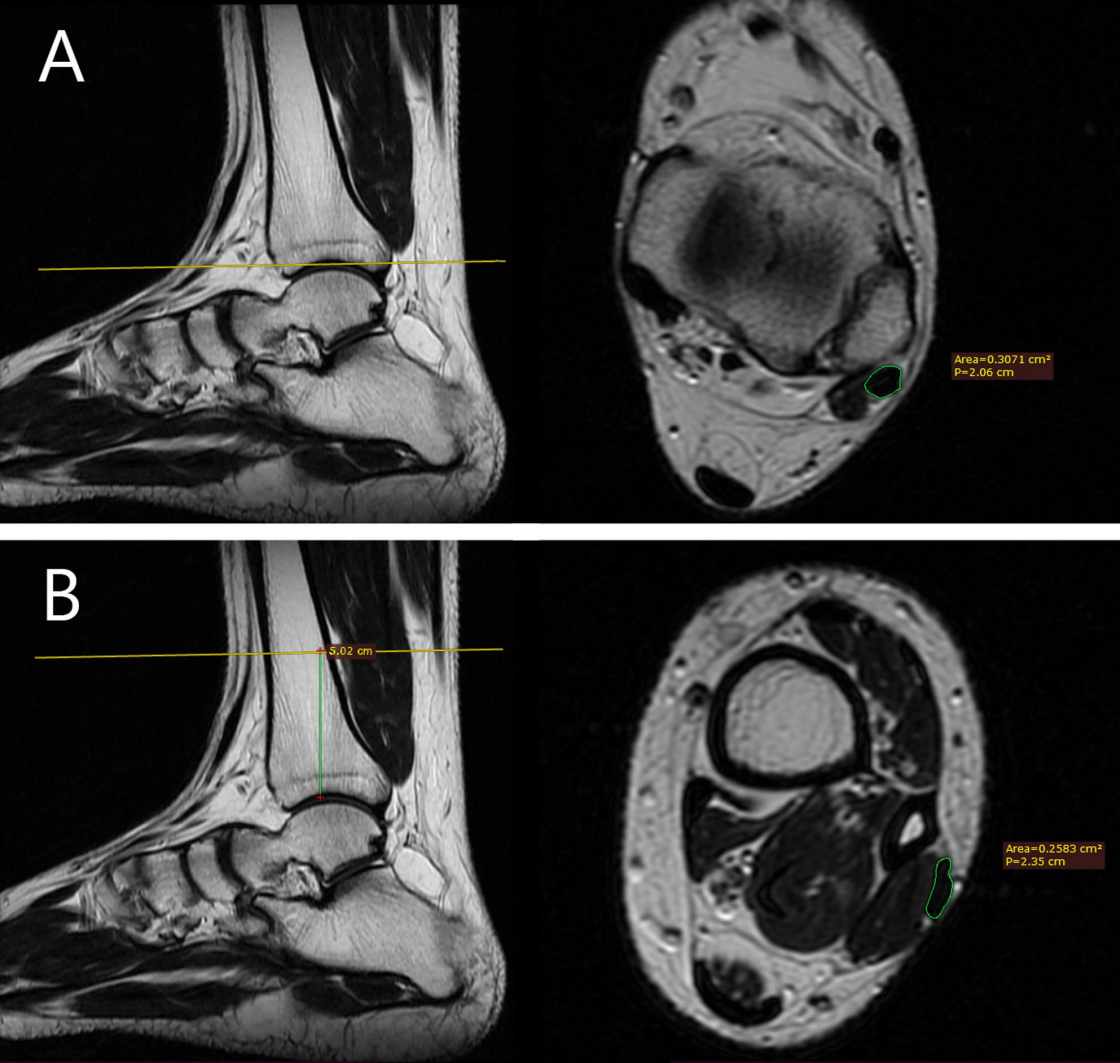
MRI images (axial image with sagittal reference for localisation) showing CSA measurement of the peroneus longus tendon at the level of the ankle joint (**A**) and 5 cm above the ankle joint (**B**)

For patients in the PLT group, additional MRI ankle screening was conducted with T2- weighted axial and coronal sequences to assess the distal morphology and CSA of the peroneus longus tendon. CSA was measured at two anatomical sites (Fig. 2):

1. At the level of the ankle joint line.
2. At the level 5 cm proximal to the ankle joint line.

Measurements were taken from axial slices with correlation to sagittal planes to maintain anatomical precision. The ankle joint level and the point 5 cm proximal to it were chosen due to their consistent tendon morphology and reliable visualisation on axial MRI slices. Previous studies suggest that the distal portion of the peroneus longus tendon maintains a stable cross-sectional profile, minimising variability and improving reproducibility of CSA measurements [29].

Following radiological evaluation, all patients were scheduled for arthroscopic ACL reconstruction surgery by the operative team.

### Surgical procedure

All procedures were performed by a single surgical team under spinal or general anaesthesia, with a high-thigh pneumatic tourniquet applied. Diagnostic arthroscopy was first performed via standard anteromedial and anterolateral portals to confirm complete ACL tear and evaluate for associated intra-articular pathology.

In the HT group, the ipsilateral semitendinosus (ST) and gracilis (GT) tendons were harvested via a medial approach and fashioned into a quadrupled graft. In the PLT group, the ipsilateral tendon was harvested from the lateral compartment of the distal leg and fashioned into a tripled graft.

Prepared grafts were sized intraoperatively using a calibrated cylindrical sizing block in 0.5 mm increments. The final graft diameter was defined as the smallest tunnel through which the graft passed without resistance. The grafts were secured with proloop or infiloop titanium loop or helysis PLDLA-bTCP (poly-L-co-DL-lactic acid-beta tricalcium phosphate) for femoral fixation and helysis titanium or PLDLA-bTCP interference screws for tibial fixation (Sironix Division, Healthium Medtech, India) depending on patient affordability, nature of the ACL rupture, and patient preference. Triclosan coated antimicrobial sutures trusynth plus neo were used to close the incision sites. Postoperative pain management and rehabilitation were carried out as planned.

### Outcome measures

The primary outcome was the correlation between MRI-derived CSA and intraoperative graft diameter for each graft type. Secondary objectives included determination of CSA cut- off values for predicting grafts with diameters ≥ 8 mm, which are considered biomechanically sufficient for ACL reconstruction.

### Statistical analysis

All patient data collected during the study were initially compiled using Microsoft Excel for structured entry and preliminary verification. The finalized dataset was then imported into IBM SPSS Statistics v25 (IBM Corp., Armonk, NY) for comprehensive statistical analysis. All evaluations were conducted within a 95% confidence interval, and a p-value < 0.05 was considered statistically significant.

Normality of continuous variables was assessed using the Shapiro–Wilk test to determine suitability for Pearson’s correlation. Non-normally distributed data were analysed using Spearman’s (ρ). There were no missing data encountered during data entry or analysis. AUC values are presented with corresponding 95% confidence intervals to reflect diagnostic precision. Effect size estimates (r²) were computed to quantify the proportion of variance in graft diameter explained by CSA measurements. All statistical models were evaluated for underlying assumptions; no violations were noted.

Descriptive statistics were used to summarize baseline characteristics and measurement distributions. The relationship between MRI-derived cross-sectional area (CSA) and intraoperative graft diameter was evaluated using Pearson’s correlation coefficient (r) for normally distributed variables and Spearman’s rho (ρ) for non-parametric data.

To assess the diagnostic utility of CSA in predicting autografts ≥ 8 mm in diameter, receiver operating characteristic (ROC) curves were generated. Sensitivity, specificity, and area under the curve (AUC) values were reported for each graft type and anatomical measurement level to determine the predictive validity of MRI.

## Results

### Descriptive statistics

Fifty-two patients were included and equally distributed between the hamstring tendon (HT) and peroneus longus tendon (PLT) groups (*n* = 26 each). All underwent preoperative MRI assessment, followed by intraoperative measurement of graft diameter after standard graft preparation.

### Hamstring tendon (HT) group

The mean age was 30.19 ± 5.10 years (range 21–45), and 76.9% were male. Graft reconstruction was slightly more frequent on the left side (53.8%). Preoperative MRI measurements included the cross-sectional area (CSA) of the gracilis (GT) and semitendinosus (ST) tendons, both individually and combined, assessed at:

- The knee joint line, and.
- The level of maximum anteroposterior diameter of the medial femoral condyle (MFC).

Following tendon harvesting and quadrupling, the mean intraoperative graft diameter was 8.29 ± 0.72 mm (range: 7.5–10.0 mm).

### Peroneus longus tendon (PLT) group

The PLT group had a mean age of 29.50 ± 8.01 years (range 18–47) with an equal sex distribution (13 male, 13 female) and symmetrical side distribution (13 left, 13 right knees). Preoperative MRI CSA was recorded at two anatomical sites:

- The ankle joint level, and.
- 5 cm proximal to the ankle joint.

Post-harvest, the tendon was tripled, and the median intraoperative graft diameter was 8.0 mm (range 7.5–10.5 mm).

Demographic characteristics, tendon CSA values at specified anatomical landmarks, and intraoperative graft diameters for both the hamstring and peroneus longus groups are summarised in Table 1.

**Table 1:** Descriptive statistics for hamstring and peroneus longus groups

Parameter	Mean ± SD	Range (Min–Max)
*A. Hamstring tendon group (n = 26)*		
Age (years)	30.19 ± 5.10	21.00–45.00
Gracilis CSA at knee joint (mm²)	7.45 ± 2.00	4.04–12.76
Semitendinosus CSA at knee joint (mm²)	11.43 ± 2.91	7.33–18.97
GT + ST combined CSA at knee joint (mm²)	18.89 ± 4.49	11.37–31.73
Gracilis CSA at MFC (mm²)	7.55 ± 1.71	3.17–11.75
Semitendinosus CSA at MFC (mm²)	12.39 ± 2.99	7.36–19.31
GT + ST combined CSA at MFC (mm²)	19.94 ± 4.18	11.44–31.06
Intraoperative graft diameter (mm)	8.29 ± 0.72	7.50–10.00
*B. Peroneus longus tendon group (n = 26)*		
Age (years)	29.50 ± 8.01	18.00–47.00
PLT CSA at ankle joint (mm²)	20.00 ± 7.15	7.53–42.46
PLT CSA 5 cm proximal (mm²)	19.83 ± 6.29	8.05–32.99
Intraoperative graft diameter (mm)	8.15 ± 0.77	7.50–10.50

### Correlation analysis

#### Hamstring tendon (HT) group

Statistically significant positive correlations were observed between MRI-derived CSA and intraoperative graft diameter:

- GT + ST CSA at the knee joint: *r* = 0.639; *p* = 0.0004.
- GT + ST CSA at the MFC level: *r* = 0.782; *p* < 0.0001.

A strong intercorrelation was also noted between CSA at the two levels: *r* = 0.793; *p* < 0.0001.

### Peroneus longus tendon (PLT) group

Spearman’s rho analysis revealed:

- CSA at the ankle joint: *r* = 0.594; *p* = 0.001.
- CSA 5 cm above the ankle joint: *r* = 0.541; *p* = 0.004.

A moderate intercorrelation was noted between CSA values at the two levels: *r* = 0.277; *p* = 0.171.

These findings confirm that MRI-based CSA measurements show predictive relationships with graft diameters in both tendon groups, particularly at the MFC and ankle joint levels, respectively (Table 2).

**Table 2:** Correlation analysis of MRI CSA values with intraoperative graft diameter

Comparison	Correlation coefficient (*r*)	*p*-value
*A. Hamstring Tendon Group (n = 26)*		
GT + ST CSA at knee vs. intraoperative diameter	0.639	0.0004
GT + ST CSA at MFC vs. intraoperative diameter	0.782	< 0.0001
GT + ST CSA at knee vs. GT + ST CSA at MFC	0.793	< 0.0001
*B. Peroneus Longus Tendon Group (n = 26)*		
PLT CSA at ankle joint vs. PLT CSA 5 cm above	0.277	0.171
PLT CSA at ankle joint vs. intraoperative diameter	0.594	0.001
PLT CSA 5 cm above ankle vs. intraoperative diameter	0.541	0.004

### ROC curve and cut-off analysis

**Fig. 3:**
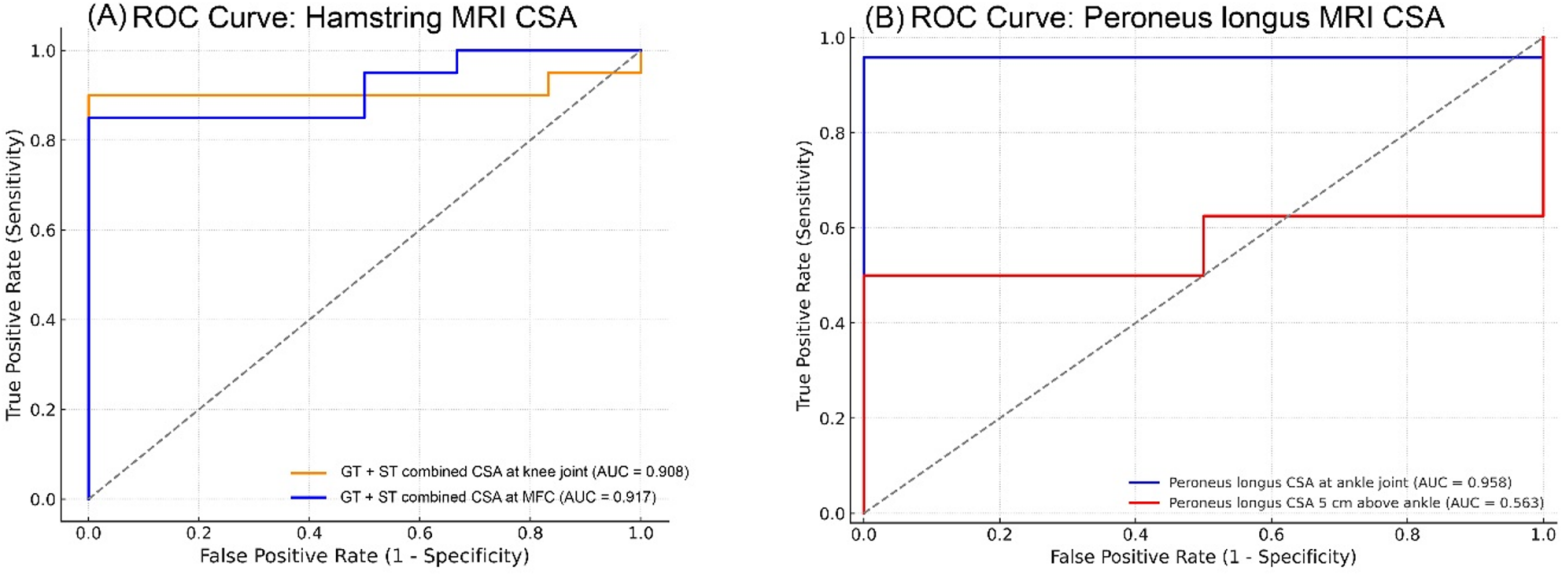
Receiver operating characteristic (ROC) curves comparing the diagnostic performance of MRI CSA values for predicting grafts ≥ 8 mm in both the hamstring (**A**) and peroneus longus (**B**) groups. Curves represent sensitivity versus 1-specificity at various CSA thresholds

To evaluate diagnostic performance, receiver operating characteristic (ROC) curves were generated for both graft types. The area under the curve (AUC), optimal cut-off values, sensitivity, specificity, and associated 95% confidence intervals (CIs) were calculated to assess the discriminatory ability of MRI-based CSA values to predict grafts ≥ 8 mm in diameter. Figure 3 shows the ROC curve for both HT and PLT groups.

### Hamstring tendon (HT) group

Both sites showed excellent diagnostic accuracy, with the MFC level slightly outperforming the knee joint line. No formal statistical test (e.g., DeLong’s test) was performed to compare these AUCs; this comparison is descriptive only.

- MFC Level: AUC = 0.917 (95% CI: 0.783–1.000); Cut-off ≥ 19.15 mm²; Sensitivity: 85%; Specificity: 100%.
- Knee joint line: AUC = 0.908 (95% CI: 0.771–1.000; Cut-off ≥ 17.13 mm²; Sensitivity: 90%; Specificity: 100%.

Both sites demonstrated excellent discriminative power, with overlapping but high-confidence intervals, confirming robustness. The MFC level showed slightly higher AUC but with comparable CIs, suggesting both measurements are valid, but the MFC site may provide greater precision.

#### Peroneus longus tendon (PLT) group

The ankle joint level demonstrated markedly superior diagnostic value, while the proximal site showed poor discrimination (AUC = 0.563), indicating limited reliability.

- Ankle Joint Level: AUC = 0.958 (95% CI: 0.878–1.000); Cut-off ≥ 12.84 mm²; Sensitivity: 95.8%; Specificity: 100%.
- 5 cm Above Ankle Joint: AUC = 0.563 (95% CI: 0.353–0.772); Cut-off ≥ 19.98 mm²; Sensitivity: 50%; Specificity: 100%.

The ankle joint level CSA showed excellent predictive strength, with a high AUC and a narrow CI close to the ideal of 1.0. In contrast, the proximal measurement exhibited poor discrimination, with a wide CI crossing 0.5, indicating limited clinical value. The statistically significant gap between these AUCs underscores the ankle joint as the optimal site for PLT assessment.

The diagnostic performance of MRI-derived CSA in predicting grafts ≥ 8 mm is summarised in Table 3.

**Table 3:** MRI-based CSA cut-off values for predicting ≥ 8 mm graft diameter

Parameter	Cut-off (mm²)	Sensitivity (%)	Specificity (%)
GT + ST combined MRI CSA at level of knee joint	17.13	90.0	100
GT + ST combined MRI CSA at maximum anteroposterior diameter of medial femoral condyle (MFC)	19.15	85.0	100
Peroneus longus MRI CSA at level of ankle joint	12.84	95.8	100
Peroneus longus MRI CSA 5 cm above level of ankle joint	19.98	50.0	100

### Interpretation of key findings

MRI-based CSA measurements showed robust and statistically significant correlations with intraoperative graft diameters across both tendon groups. The maximum AP diameter at the MFC (HT group) and the ankle joint (PLT group) provided the most reliable predictions for grafts ≥ 8 mm. The defined cut-offs for each group demonstrated excellent sensitivity and specificity, supporting the clinical utility of MRI morphometry for preoperative graft selection in ACL reconstruction.

## Discussion

### Overview and purpose

This study aimed to determine whether preoperative MRI-derived cross-sectional area (CSA) measurements of the semitendinosus–gracilis complex and the peroneus longus tendon could predict intraoperative graft diameter in ACL reconstruction. Graft diameter is a key determinant of reconstruction success, with sub-8 mm grafts associated with increased failure risk and revision rates. In this context, establishing a reliable, non-invasive imaging method to anticipate graft adequacy preoperatively has strong clinical relevance. This investigation was designed to improve surgical readiness, enhance patient counselling, and minimize intraoperative uncertainty by identifying predictive anatomical thresholds.

### Correlative findings and diagnostic insights

In both graft groups, MRI-based CSA measurements demonstrated significant positive correlations with intraoperative graft diameters. Among patients undergoing hamstring grafting, the highest correlation was found for combined gracilis and semitendinosus CSA measured at the medial femoral condyle (MFC) level (*r* = 0.782; *p* < 0.001), followed by the knee joint line (*r* = 0.639; *p* = 0.0004). The inter-correlation between these two anatomical sites (*r* = 0.794) supports the internal reliability of the imaging protocol. These results confirm that CSA assessments at anatomically consistent points—particularly the MFC level—serve as robust predictors of final graft diameter.

In the peroneus longus group, CSA measured at the ankle joint also showed strong correlation with intraoperative graft size (*r* = 0.594; *p* = 0.001), exceeding the correlation found at 5 cm proximal to the joint (*r* = 0.541; *p* = 0.004). These findings emphasize the value of site-specific tendon imaging, reflecting anatomical consistency in the distal segment of the peroneus longus and the confounding tapering observed more proximally.

### ROC-based predictive thresholds

Receiver Operating Characteristic (ROC) analysis quantified the diagnostic value of CSA metrics in predicting grafts of clinically acceptable size (≥ 8 mm). In the hamstring group, CSA measured at the MFC yielded an AUC of 0.917 (95% CI: 0.783–1.000) with a cut-off of ≥ 19.15 mm², achieving 85% sensitivity and 100% specificity. CSA measured at the knee joint line level yielded an AUC of 0.908 (95% CI: 0.771–1.000) with a cut-off of ≥ 17.13 mm² produced slightly higher sensitivity (90%) with equally perfect specificity (100%). The specificity of both cut-offs suggests that CSA values above these thresholds reliably identify patients likely to yield adequate grafts.

In the peroneus longus group, CSA at the ankle joint emerged as the most reliable predictor, with a cut-off of ≥ 12.84 mm² delivering a high AUC (0.958), 95.8% sensitivity, and 100% specificity. In contrast, the 5 cm proximal site offered poor discriminatory power (AUC = 0.563), reaffirming the ankle joint as the preferred measurement site.

### Comparison with previous literature

Our findings align well with previous studies that have evaluated MRI CSA as a predictive tool for hamstring graft adequacy. Bhamare et al. (2022) [17] reported strong correlation (*r* = 0.838) using CSA values at the joint line and MFC, proposing a threshold of 17.5 mm². Hollnagel et al. (2019) [13] also noted strong correlations (*r* ~ 0.70) and defined a cut-off of 18.8 mm² for 1.5T MRIs. Similar cut-offs were described by Leiter et al. (2016) [28] and Wernecke et al. (2011) [30], who all reinforced the MFC as the most consistent site. The present study corroborates these observations, while also offering a slight refinement—identifying the MFC CSA as having higher AUC and correlation than the joint line, thus supporting its use as a primary metric in clinical settings.

No prior studies have systematically evaluated the predictive utility of MRI CSA in peroneus longus tendons. This investigation is the first to propose imaging-derived CSA thresholds for PLT grafts in ACL reconstruction, establishing both anatomical sites and diagnostic values. The identification of a reliable cut-off (≥ 12.84 mm²) with excellent sensitivity and specificity fills a significant gap in graft planning for cases where hamstrings are unsuitable.

## Clinical implications

The introduction of CSA-based prediction can transform preoperative planning in ACL surgery. Anticipating graft adequacy enables surgeons to counsel patients accurately and prepare for alternate strategies such as graft augmentation, allograft planning, or dual graft techniques. This is particularly relevant in athletes or skeletally immature patients, where small grafts are biomechanically disadvantageous.

The anatomical CSA thresholds identified here—19.15 mm² at the MFC (HT group) and 12.84 mm² at the ankle joint (PLT group)—can serve as objective, reproducible indicators to streamline surgical workflows and reduce intraoperative surprises.

### Strengths and limitations

This study features several methodological strengths. All MRI measurements were performed by a single experienced musculoskeletal radiologist using consistent imaging parameters and anatomical landmarks. Likewise, a single surgical team ensured uniform graft harvest and sizing techniques. These controls enhanced measurement reliability and internal validity.

Importantly, this is the first study to define MRI CSA thresholds for PLT autografts in ACL reconstruction. While prior research has focused on hamstrings, the novel inclusion of peroneus longus widens the scope of individualized graft planning.

Nonetheless, limitations exist. The sample size (*n* = 26 per group) restricts generalizability, and all imaging measurements were made by a single observer without inter-observer reliability assessment. Additionally, anthropometric variables including height, weight, and BMI were not recorded in this study. Given that these factors are known to correlate with tendon dimensions, their inclusion in future studies would further strengthen predictive models. Furthermore, intra-observer reliability was not formally assessed; the manual selection of axial slices and drawing of regions of interest (ROI) by the same radiologist over time may introduce measurement variability, and the absence of intra-class correlation coefficient (ICC) testing is acknowledged as a limitation Additionally, while ROC-derived thresholds performed well in this cohort, external validation remains necessary. This study also did not assess long-term outcomes such as graft survival, re-rupture, or patient-reported metrics, which would provide further clinical depth.

### Future directions

Larger, multi-institutional studies are warranted to validate the cut-off thresholds proposed here. Advanced MRI methods such as 3D reconstruction or machine learning–assisted morphometry may further refine prediction accuracy. Finally, stratification by demographic factors such as sex, BMI, and athletic level may allow more personalized graft selection.

## Conclusion

This study demonstrated that preoperative MRI-based cross-sectional area (CSA) measurements are reliable predictors of intraoperative graft diameter in ACL reconstruction.

It supports the use of MRI-derived CSA as a non-invasive, preoperative tool to guide graft selection, reduce intraoperative uncertainty, and improve surgical planning. The study also introduces novel predictive metrics for peroneus longus tendons—an area previously underexplored in the literature. While the results are promising, further validation in larger, multi-center studies is recommended to confirm generalizability and clinical applicability.

## Data Availability

The datasets used and analyzed during the current study are available from the corresponding author upon reasonable request.

## Declarations

### Patient and Public Involvement

Patients or members of the public were not involved in the design, conduct, reporting, or dissemination of this research.

### Equity, Diversity, and Inclusion Statement

Participants were recruited regardless of gender, ethnicity, or socioeconomic status. Although subgroup analysis was not performed, the sampling process was inclusive of a general orthopaedic surgical population.

## Abbreviations

MRI: Magnetic resonance imaging
CSA: Cross-sectional area
ACL: Anterior cruciate ligament
HT: Hamstring tendon
ROC: Receiver operating characteristic
PLT: Peroneus longus tendon
ACLR: ACL reconstruction
QT: Quadriceps tendon
BPTB: Bone–patellar tendon–bone
ST: semitendinosus tendon
GT: Gracilis tendon
AP: Anteroposterior
PLDLA-bTCP: Poly-L-co-DL-lactic acid-beta tricalcium phosphate
AUC: Area under the curve
MFC: Medial femoral condyle

## References

[1] Bosco DS, Raj PAP, Krishnan P, Narayanan H, Adisegaran A. Prospective evaluation of functional outcome in anterior cruciate ligament reconstruction with peroneus longus graft: a hospital-based study. Int J Res Orthop. 2025;11(2):308–13. 10.18203/issn.2455-4510.IntJResOrthop20250451.

[2] Sauer SACL Reconstruction. Current concepts. Cham (Switzerland). Springer; 2024. . 10.1007/978-3-031-69000-6.

[3] Piedade SR, Leite Arruda BP, de Vasconcelos RA, Parker DA, Maffulli N. Rehabilitation following surgical reconstruction for anterior cruciate ligament insufficiency: what has changed since the 1960s? – state of the art. J ISAKOS. 2023;8(3):153–62. 10.1016/j.jisako.2022.10.001. 36410671 10.1016/j.jisako.2022.10.001

[4] Shino K. Essence of anterior cruciate ligament: scientific evidence and clinical practice of ACL. Singapore: Springer Nature Singapore; 2023. 10.1007/978-981-99-6536-6.

[5] Chang CB, Seong SC, Kim TK. Preoperative magnetic resonance assessment of patellar tendon dimensions for graft selection in anterior cruciate ligament reconstruction. Am J Sports Med. 2009;37(2):376–82. 19036719 10.1177/0363546508324971

[6] Persson A, Fjeldsgaard K, Gjertsen JE, et al. Increased risk of revision with hamstring tendon grafts compared with patellar tendon grafts after ACL reconstruction: a study of 12,643 patients from the Norwegian Cruciate Ligament Registry, 2004–2012. Am J Sports Med. 2014;42(2):285–91. 10.1177/0363546513511419. 24322979 10.1177/0363546513511419

[7] Gifstad T, Foss OA, Engebretsen L, et al. Lower risk of revision with patellar tendon autografts compared with hamstring autografts: a registry study based on 45,998 primary ACL reconstructions in Scandinavia. Am J Sports Med. 2014;42(10):2319–28. 10.1177/0363546514548164. 25201444 10.1177/0363546514548164

[8] Maffulli N, Osti L. ACL stability, function, and arthritis: what have we been missing? Orthopedics. 2013;36(2):90–2. 10.3928/01477447-20130122-02. 23379658 10.3928/01477447-20130122-02

[9] Samuelsen BT, Webster KE, Johnson NR, Hewett TE, Krych AJ. Hamstring autograft versus patellar tendon autograft for ACL reconstruction: is there a difference in graft failure rate? A meta-analysis of 47,613 patients. Clin Orthop Relat Res. 2017;475(10):2459–68. 10.1007/s11999-017-5278-9. 28205075 10.1007/s11999-017-5278-9 PMC5599382

[10] Magnussen RA, Lawrence JTR, West RL, Toth AP, Taylor DC, Garrett WE. Graft size and patient age are predictors of early revision after anterior cruciate ligament reconstruction with hamstring autograft. Arthroscopy. 2012;28(4):526–31. 10.1016/j.arthro.2011.11.024. 22305299 10.1016/j.arthro.2011.11.024

[11] Mariscalco MW, Flanigan DC, Mitchell J, et al. The influence of hamstring autograft size on patient-reported outcomes and risk of revision after ACL reconstruction: a MOON cohort study. Arthroscopy. 2013;29(12):1948–53. 10.1016/j.arthro.2013.08.025. 24140144 10.1016/j.arthro.2013.08.025 PMC3844091

[12] Park SY, Oh H, Park S, Lee JH, Lee SH, Yoon KH. Factors predicting hamstring tendon autograft diameters and resulting failure rates after ACL reconstruction. Knee Surg Sports Traumatol Arthrosc. 2013;21(5):1111–8. 22688502 10.1007/s00167-012-2085-4

[13] Hanna A, Hollnagel K, Whitmer K, et al. Reliability of MRI prediction of ACL autograft size and comparison of radiologist and orthopaedic surgeon predictions. Orthop J Sports Med. 2019;7(12):2325967119889593. 10.1177/2325967119889593. 31858015 10.1177/2325967119889593 PMC6913056

[14] Grawe BM, Williams PN, Burge A, et al. ACL reconstruction with autologous hamstring: can preoperative MRI accurately predict graft diameter? Orthop J Sports Med. 2016;4(5):2325967116646360. 10.1177/2325967116646360. 27294166 10.1177/2325967116646360 PMC4887876

[15] Magnussen RA, Lawrence JTR, West RL, Toth AP, Taylor DC, Garrett WE. Graft size and patient age are predictors of early revision after ACL reconstruction with hamstring autograft. Arthroscopy. 2012;28(4):526–31. 10.1016/j.arthro.2011.11.024. 22305299 10.1016/j.arthro.2011.11.024

[16] Mariscalco MW, Flanigan DC, Mitchell J, et al. The influence of hamstring autograft size on outcomes and risk of revision after ACL reconstruction: a MOON cohort study. Arthroscopy. 2013;29(12):1948–53. 10.1016/j.arthro.2013.08.025. 24140144 10.1016/j.arthro.2013.08.025 PMC3844091

[17] Bhamare DS, Sirasala S, Jivrajani P, Nair A, Taori S. Preoperative MRI assessment of hamstring tendons to predict the quadruple hamstring graft diameter in ACL reconstruction. Cureus. 2022;14(1):e21753. 10.7759/cureus.21753. 35251824 10.7759/cureus.21753 PMC8890813

[18] Albarello JC, Laett CT, de Palma AMS, et al. Associated ACL reconstruction and meniscal repair do not affect the evolution of isokinetic parameters in professional athletes: a prospective study with a one-year follow-up. Muscles Ligaments Tendons J. 2024;14(3):450–7. 10.32098/mltj.03.2024.08.

[19] Lemos T, Albarello JCS, Silva SC, Mozella AP. Quadriceps force fluctuation during maximal isometric contraction is altered in ACL injury and is associated with lower limb functional performance. Muscles Ligaments Tendons J. 2024;14(3):499–506. 10.32098/mltj.03.2024.13.

[20] Movahedinia M, Movahedinia S, Hosseini S, et al. Prediction of hamstring tendon autograft diameter using preoperative measurements with different cut-offs between genders. J Exp Orthop. 2023;10(1):4. 10.1186/s40634-023-00569-0. 36680691 10.1186/s40634-023-00569-0 PMC9867787

[21] Bickel BA, Fowler TT, Mowbray JG, Adler B, Klingele K, Phillips G. Preoperative MRI cross-sectional area for the measurement of hamstring autograft diameter for reconstruction of the adolescent ACL. Arthroscopy. 2008;24(12):1336–41. 10.1016/j.arthro.2008.07.012. 19038703 10.1016/j.arthro.2008.07.012

[22] Agrawal S, Hegde AS, Rao BS, et al. Peroneus longus tendon autograft for primary arthroscopic reconstruction of the ACL. Muscles Ligaments Tendons J. 2023;13(2):252–8. 10.32098/mltj.02.2023.08.

[23] Rhatomy S, Hartoko L, Setyawan R, et al. Single bundle ACL reconstruction with peroneus longus tendon graft: 2-years follow-up. J Clin Orthop Trauma. 2020;11(Suppl 3):S332–6. 10.1016/j.jcot.2019.09.004. 32523289 10.1016/j.jcot.2019.09.004 PMC7275277

[24] Goyal T, Paul S, Choudhury AK, Sethy SS. Full-thickness peroneus longus tendon autograft for ACL reconstruction in multi-ligament injury and revision cases. Eur J Orthop Surg Traumatol. 2023;33(1):21–7. 10.1007/s00590-021-03145-3. 34698925 10.1007/s00590-021-03145-3

[25] Bosco DS, Raj PAP, Krishnan P, Narayanan H, Adisegaran A. Prospective evaluation of functional outcome in ACL reconstruction with peroneus longus graft: a hospital-based study. Int J Res Orthop. 2025;11(2):308–13. 10.18203/issn.2455-4510.IntJResOrthop20250451.

[26] Hodges CT, Shelton TJ, Bateni CP, et al. The medial epicondyle of the distal femur is the optimal location for MRI measurement of semitendinosus and gracilis tendon cross-sectional area. Knee Surg Sports Traumatol Arthrosc. 2019;27(11):3498–504. 10.1007/s00167-019-05421-6. 30809723 10.1007/s00167-019-05421-6

[27] Mansur H, Estanislau G, de Noronha M, Marqueti RC, Fachin-Martins E, Durigan JLQ. Intra- and inter-rater reliability for the measurement of the cross-sectional area of ankle tendons assessed by MRI. Acta Radiol. 2022;63(4):481–8. 10.1177/02841851211003284. 34247515 10.1177/02841851211003284

[28] Leiter J, Elkurbo M, McRae S, Chiu J, Froese W, MacDonald P. Using pre-operative MRI to predict intraoperative hamstring graft size for ACL reconstruction. Knee Surg Sports Traumatol Arthrosc. 2017;25(1):229–35. 10.1007/s00167-016-4205-z. 27440154 10.1007/s00167-016-4205-z

[29] Pipino G, Tomasi E, Mardones R, et al. Rehabilitation after anterior cruciate ligament reconstruction: dry land vs aquatic rehabilitation. Muscles Ligaments Tendons J. 2023;13(3):421–9. 10.32098/mltj.03.2023.10.

[30] Wernecke G, Harris IA, Houang MTW, Seeto BG, Chen DB, MacDessi SJ. Using MRI to predict adequate graft diameters for autologous hamstring double-bundle ACL reconstruction. Arthroscopy. 2011;27(8):1055–9. 10.1016/j.arthro.2011.02.035. 21704471 10.1016/j.arthro.2011.02.035

